# Ischemic Gastropathic Ulcer Mimics Gastric Cancer

**DOI:** 10.1155/2016/9745854

**Published:** 2016-08-10

**Authors:** Saleh Daher, Ziv Lahav, Ayman Abu Rmeileh, Meir Mizrahi, Tawfik Khoury

**Affiliations:** ^1^Division of Gastroenterology and Hepatology, Department of Medicine, Hebrew University-Hadassah Medical Center, P.O. Box 12000, Ein Kerem, 91120 Jerusalem, Israel; ^2^Department of Internal Medicine, Hebrew University-Hadassah Medical Center, P.O. Box 12000, 91120 Jerusalem, Israel; ^3^Division of Gastroenterology and Hepatology, Advanced Endoscopy Center, Beth Israel Deaconess Medical Center, Harvard Medical School, 330 Brookline Avenue, Stoneman 458, Boston, MA 02215, USA

## Abstract

Gastric ulcer due to mesenteric ischemia is a rare clinical finding. As a result, few reports of ischemic gastric ulcers have been reported in the literature. The diagnosis of ischemic gastropathy is seldom considered in patients presenting with abdominal pain and gastric ulcers. In this case report, we describe a patient with increasing abdominal pain, weight loss, and gastric ulcers, who underwent extensive medical evaluation and whose symptoms were resistant to medical interventions. Finally he was diagnosed with chronic mesenteric ischemia, and his clinical and endoscopic abnormalities resolved after surgical revascularization of both the superior mesenteric artery and the celiac trunk.

## 1. Introduction

Chronic mesenteric ischemia classically presents as “abdominal angina,” characterized by generalized postprandial abdominal pain lasting up to 3 hours, as well as weight loss and upper-abdominal bruit. Symptoms are not specific and often mistakenly attributed to other gastrointestinal etiologies, such as peptic ulcer or gallstones. Gastric ischemia is not commonly encountered because of the rich collateral blood supply to the stomach, making gastric ulceration from ischemia a rare condition [1–3]. Gastric ischemia manifested as gastric ulcer might result from localized or diffuse vascular insufficiency caused by etiologies such as systemic hypotension, vasculitis, or localized thromboembolism. However,* Helicobacter pylori (HP) *infection is considered to be the major cause of peptic ulcer disease, and the use of nonsteroidal anti-inflammatory drugs (NSAIDs) accounts for the majority of the remainder [4]. Herein we report a longstanding case of chronic mesenteric ischemia where an* HP-*negative gastric ulcer and not associated with the use of NSAIDs was detected in the initial evaluation. The patient's complaints ultimately responded to revascularization surgery, with resolution of his non-NSAID, non-*HP *gastric ulcers.

## 2. Case Report

A 46-year-old male presented with progressive, mostly postprandial abdominal pain, and significant weight loss of almost 44 lb. in a six-month period.

His medical history was notable for heavy smoking for the past 30 years and cholelithiasis. His medications included 40 mg esomeprazole once a day.

Four months prior to presentation, he underwent an esophagogastroduodenoscopy (EGD) to evaluate similar complaints and was diagnosed with peptic ulcer disease (PUD) with positive* HP*. No improvement followed a course of a high dosage of esomeprazole and* Helicobacter* eradication therapy.

On admission the patient was stable, with a heart rate of 65 beats/min., blood pressure of 110/60 mmHg, and oxygen saturation of 98%. His physical examination was unremarkable except for epigastric tenderness and bilateral temporal and extremities wasting. Laboratory tests revealed normal CBC, kidney, and liver function. His C-reactive protein and erythrocyte sedimentation rate were also unremarkable.

**Figure 1:**
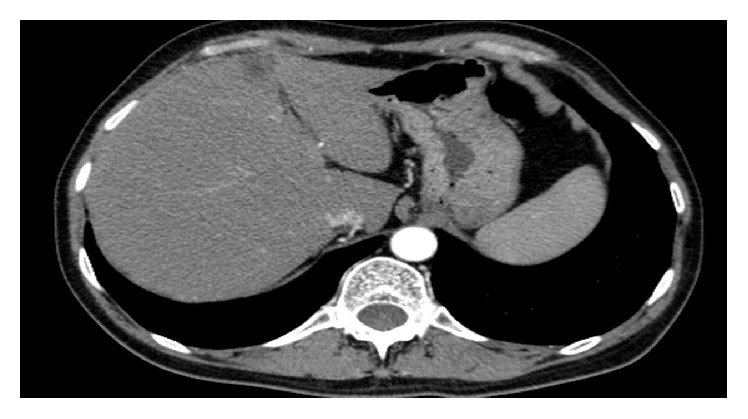
Computed tomography (CT) revealed a thickened gastric wall mainly involving the pyloric region.

**Figure 2:**
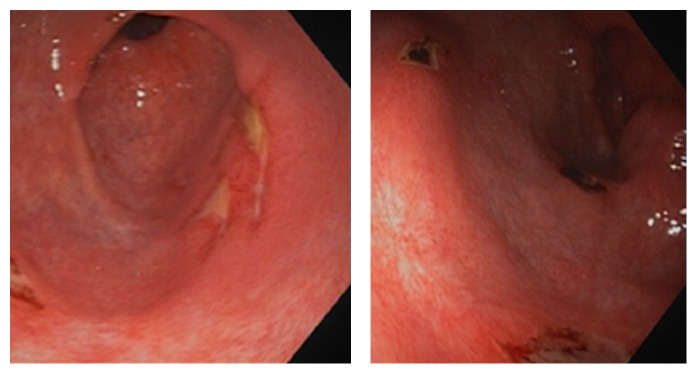
EGD showed multiple gastric ulcers in the body of the stomach, fundus, and pylorus.

Upon admission, a review of a computed tomography (CT) scan was done in an outpatient setting and revealed fatty liver, thickened gastric wall (Figure 1), and a hypodense area in the stomach; however no revision of the gastrointestinal arterial vasculature was done at that time. The patient underwent second EGD that revealed mildly erythematous antral mucosa. An immunohistochemical stain for HP was negative. Random gastric biopsies showed mild chronic active gastritis. The patient was discharged with the impression that he is suffering from active nonspecific gastritis. The dosage of esomeprazole was escalated to 40 mg twice a day. Due to the continuity of his symptoms, a third EGD was performed that showed hyperemic erythematous gastric mucosa with few longitudinal ulcerations that were located on the greater curvature on a preantral location. The pathology examination from gastric biopsies revealed a single focus of markedly atypical glands with necrotic material in the lumen, suspicious of a malignancy. A week later, a fourth EGD showed large ulcerations on the preantral greater and lesser curvatures (Figure 2) with hyperemic intervening mucosa. A pathological examination showed acute gastritis and duodenitis, reactive atypia, negative stain for* HP*, and no signs of intestinal metaplasia or malignancy.

**Figure 3:**
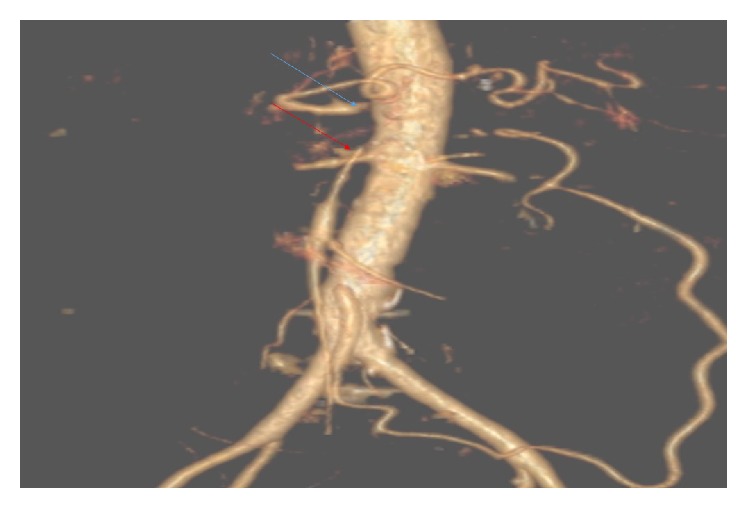
Computed tomography (CT) angiogram shows stenosis of the SMA and celiac trunk origins.

Six weeks later, he was admitted again due to worsening of epigastric pain and further weight loss, overall losing 66 lbs. over 9 months. During the third hospitalization, a diagnosis of ischemia was considered. A CT angiogram showed significant gastric pyloric wall thickening and surrounding small lymphadenopathy, several new splenic infarcts, and significant narrowing and obstruction in the origin of the superior mesenteric artery (SMA) and the origin of the celiac trunk with mild stenosis of the inferior mesenteric artery (IMA) origin (Figure 3). A vascular surgery consult was obtained and open surgical revascularization was recommended given our patient's young age and lack of comorbidities. The patient underwent a surgical bypass with expanded polytetrafluoroethylene (ePTFE) graft between the right common iliac artery and the SMA with extension graft to the hepatic artery that resulted in significant clinical improvement and weight gain. Repeat EGD following the surgical bypass revealed mild gastritis and duodenitis with resolution of the gastric ulcers.

## 3. Discussion

Gastric ulceration as a direct result of chronic mesenteric ischemia is a rare condition. The normal mesenteric arterial blood supply relies on three major branches of the abdominal aorta: the celiac trunk, the SMA, and the IMA. Most of the gastric blood supply is through the celiac artery. However, the stomach receives additional blood from a rich collateral mesenteric circulation that makes the stomach less vulnerable to ischemic insult. While an isolated stenosis of the celiac trunk is usually well tolerated, a concomitant compromised SMA blood supply can be enough to cause ischemic gastric ulcer [5–7]. The clinical presentation of ischemic gastropathy is often misinterpreted. Classic symptoms include sitophobia, postprandial abdominal pain, and weight loss [8]. However, the concomitant occurrence of more nonspecific symptoms, such as vomiting, diarrhea, and nausea, can complicate the clinical presentation [9], and the abdominal pain that is typical of mesenteric ischemia is incorrectly attributed to the presence of a gastric ulcer.

Gastric ulcers are usually attributed to more common etiologies, such as* HP* infection or NSAIDs. The irregular, ill-defined endoscopic characteristics of these gastric ulcers may also raise a suspicion of gastric malignancy, especially when weight loss is reported. Histology from a gastric ulcer usually reveals nonspecific findings of inflammation and reactive changes that usually lead to a diagnosis of gastritis [8].

Revascularization is the appropriate treatment in cases of ischemic gastropathy. This may be either by surgery or by angioplasty, with or without the use of stents [7].

The choice of the revascularization method (open versus endovascular) is generally based on patient's age and comorbidities, as well as the anatomy of vascular lesions [10–12]. For young patients without contraindications for open surgery, open surgical revascularization may be the preferred initial approach [13].

In most cases, after successful revascularization, the endoscopic findings disappear within months, while the clinical symptoms resolve within few days [14].

We have described herein a patient with recurrent postprandial abdominal pain, significant weight loss, and an HP-negative gastric ulcer without history of NSAID use. The similarity of symptoms between chronic mesenteric ischemia and gastric cancer, and the presence of gastric ulcers, led us to ruling out a malignancy, despite the fact that the clinical presentation was classic for chronic mesenteric ischemia. Finally, our patient was diagnosed with severe SMA and celiac stenosis, which most probably caused the gastric ulceration due to the concomitant compromised collateral mesenteric circulation, with complete clinical and endoscopic resolution after successful revascularization.

This case highlights the need to be aware of this occurrence. We believe that increased awareness and knowledge of this disease can result in a much faster diagnosis, specifically with the increasing availability of noninvasive imaging by CT or MRI angiography that can rapidly exclude gastrointestinal vascular incompetence [15].

In conclusion, it is extremely important to fully investigate nonhealing, ill-defined gastric ulcers for the possibility of malignancy; however, gastric ulcers that mimic gastric cancer, that are resistant to treatment, or that are* HP-*negative with no history of NSAID use should be investigated for a possible ischemic etiology, especially in patients with concomitant atherosclerotic vascular disease.
